# Optimum Conditions of Radioligand Receptor Binding Assay of Ligands of Benzodiazepine Receptors 

**Published:** 2014

**Authors:** Fatemeh Ahmadi, Sara Dabirian, Mehrdad Faizi, Sayyed Abbas Tabatabai, Davood Beiki, Soraya Shahhosseini

**Affiliations:** a *Department of Radiopharmacy, School of Pharmacy, Tehran University of Medical Sciences, Tehran, Iran. *; b *Department of Pharmaceutical Chemistry, School of Pharmacy, Shahid Behesti University of Medical Sciences, Tehran, Iran.*; c *Department of Pharmacology and Toxicology, School of Pharmacy, Shahid Beheshti University of Medical Sciences, Tehran, Iran.*; d *Research center for Nuclear Medicine, Tehran University of Medical Sciences, Tehran, Iran. *

**Keywords:** Radioligand receptor binding assay;, [3H]-flumazenil, liquid scintillation, Benzodiazepine

## Abstract

To obtain drugs which are more selective at benzodiazepine (BZD) receptors, design and synthesis of functionally selective ligands for BZD receptors is the current strategy of our pharmaceutical chemistry department. The affinity of newly synthesized ligands is assessed by radioligand receptor binding assays. Based on our previous studies, 2-phenyl-5-oxo-7-methyl-1,3, 4-oxadiazolo[a,2,3]-pyrimidine (compound A) was chosen for design and synthesis of new triazole derivatives as GABAA BZD receptor agonist. The cortical membrane of male Sprague-Dawley rats was prepared as the source of the BZD receptors. Different concentrations of membrane protein and [3H]-flumazenil were incubated at room temperature at different time periods to reach the steady-state. To saturate the receptors, increased amounts of radioligand were incubated with membrane protein. The bound and un-bound ligands were separated by centrifugation. The affinity of compound A was measured in competition studies at optimum conditions by displacement of [3H]-Flumazenil from rat cortical membrane. Based on results, the optimum conditions of radioligand receptor binding assay of benzodiazepines were 35 min incubation of ligands with 100 μg cortical membrane protein and 8.6 × 10-5 nmole 3H-flumazenil in a final volume of 0.5 mL Tris-HCl buffer (50 mM, pH 7.4) at 30 °C. The binding parameters of [3H]-flumazenil, Bmax and Kd were determined through saturation studies as 0.638 ± 0.099 pmol/mg and 1.35 ± 0.316 nM respectively. The affinity of compound A was 1.9 nM comparable with diazepam (1.53nM). This finding makes the compound an interesting lead for further optimization. Starting from this compound, new ligands were synthesized and screened *in-vitro *by competitive binding assays.

## Introduction

Radioligand binging assays are used to determine the affinity of various ligands for a receptor, the binding site density of receptor families and their subtypes in different tissues or samples, the distribution of receptors, and effects of physiological and pathological conditions on the expression of the receptors. There are two basic types of receptor binding experiments: saturation and competition. Saturation studies are used to determine the affinity of radioligand for a receptor (Kd) and the density of receptors (Bmax) in specific tissues or samples. Competition studies are used to determine the affinity of non-radioactive ligands for receptors (1). 

The pharmacological effects of benzodiazepines (BZDs) such as anxiolytic, anticonvulsant, muscle relaxant, and sedative-hypnotic properties, make them the most important GABAA receptor modulating drugs currently in clinical use. It is suggested that the specific pharmacological effects of BZDs may be mediated by binding to the BZD binding site of the central GABAA receptor (2). New BZD receptor ligands with more selective effects such as anti-anxiety, anti-seizure and fewer adverse drug reactions were synthesized in the last two decades (3-8). To assess the affinity of novel ligands for the binding site, radioligand receptor binding assays are widely utilized by investigators to quickly and inexpensively screen the affinity of ligands for the receptors *in-vitro *(9). The experiments are done at optimum conditions in order to get reproducible and reliable results. The medicinal chemistry researchers would further analyze the structure activity relationship (SAR) for new derivatives at GABAA BZD receptor and find a lead compound using the binding assay results. 

In this work, the optimum conditions of radioligand receptor binding assay to reach steady state such as incubation time, membrane protein and radioligand concentration were studied. The binding parameters of [3H]-flumazenil were determined through saturation studies using centrifugation method. The affinity of ligands (Ki) was determined through competition studies in comparison with diazepam as a standard agonist of benzodiazepine receptors. In this paper, we report the results of these studies using rat cortical membranes as the source of the BZD receptors and [3H]-flumazenil as the radioligand. 

## Experimental

*Membrane preparation*

Male Sprague-Dawley rats with weights of 300-350 g (Pasteur Institute, Tehran, Iran) were anesthetized with CO2 and then sacrificed. The cortical membrane tissue was immediately removed and homogenized for 30 s in 20 mL ice-cold Tris-HCl buffer (30 mM, pH 7.4) using a Silent S homogenizer (Heidolph, Germany) at medium speed. The homogenates were centrifuged at 600 g for 10 min using a Beckman Coulter L90K centrifuge. The resulting supernatant was centrifuged at 27000 g for 15 min. The pellet was washed 3 times with ice-cold buffer by re-suspension and re-centrifugation. The washed pellet was suspended in 20 mL buffer, incubated at 37 °C for 30 min and then centrifuged for 10 min at 27000 g. The pellet was washed once, and the final pellet was re-suspended in 30 mL Tris-HCl buffer (50 mM, pH 7.4). All of the centrifugation was performed at 4 °C (10-12). The amount of protein was estimated in the membrane preparation by the Bradford method (1976) using bovine serum albumin (BSA) as a standard (13). The membrane preparation was stored at -20 °C until it was used 1-15 days later. 

*Assay conditions*

*Incubation time*

100 μg of membrane protein was incubated with 8.6×10-5 nmole 3H-flumazenil (87Ci/mmol, Perkin-Elmer, USA Life and Analytical Science) in a final volume of 0.5 mL Tris-HCl buffer (50 mM, pH 7.4) at 30 °C. After incubation in different time periods (10, 20, 25, 30, 40 min), the contents of the tube were centrifuged at 1500 g for 4 min at 4 °C using Tomy MX-305 refrigerated centrifugation (Tomy, Japan). The supernatant was gently aspirated from the pellet. Pellet was washed by ice-cold Tris-HCl buffer, transferred to liquid scintillation vials, covered with 1mL of liquid scintillation cocktail (Maxilight, Hidex, Finland) and the activity was measured by liquid scintillation counter (Triathler multilabel tester, Hidex, Finland). Nonspecific binding (NSB) was determined in parallel assays performed in the presence of 100 μM diazepam. Total binding (TB) (receptor + radioligand) and NSB (receptor + radioligand + excess diazepam) were measured at various times of incubation (1, 14-16). 

*Receptor concentration (Zone A)*

Different concentrations of membrane proteins (50, 100, 150, 200, 250, 300 μg) in Tris-HCl buffer (50 mM, pH 7.4) were incubated with 8.6×10-5 nmole [3H]-flumazenil in total volume of 0.5 mL at 30 °C. After a 35 minute incubation period, the homogenate was centrifuged and the activity of pellet was measured as previously mentioned. TB (receptor + radioligand) and NSB (receptor + radioligand + excess diazepam) were measured at various levels of added membrane protein. Total added (TA) is the count of radioligand in absence of receptor and diazepam (16). 

*Saturation binding studies*

100 μg of membrane protein was added to Tris-HCl buffer (50 mM, pH 7.4) and incubated with seven different concentrations of [3H]-Flumazenil at 30 °C for 35 min. The incubation was terminated by the centrifugation of reaction mixture at 1500 g for 4 min at 4 °C. The activity of pellet was measured as previously mentioned. TB (receptor + radioligand), NSB (receptor + radioligand + excess diazepam), and specific binding (SB) (SB=TB-NSB) were measured at various radioligand concentrations (1, 9). 

*Competition binding studies*

**Figure 1:**
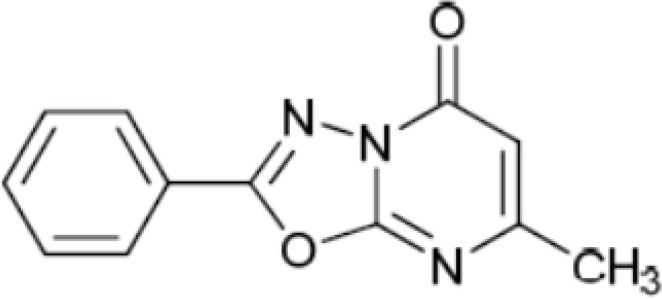
The structure of 2-phenyl-5-oxo-7-methyl-1,3, 4-oxadiazolo [a, 2, 3]-pyrimidine (compound A).

100 μg of membrane protein in Tris-HCl buffer (50 mM, pH 7.4) was incubated with 8.6×10-5 nmole [3H]-Flumazenil and increasing amount of 2-phenyl-5-oxo-7-methyl-1,3,4-oxadiazolo[a,2,3]-pyrimidine, (compound A, Figure 1) in a final volume 0.5mL at 30 °C for 35 min (17). After incubation, the assay was terminated by centrifugation (1500 g, 4°C, 4 min). The activity of pellet was measured as previously mentioned. Binding (receptor + radioligand) and NSB (receptor + radioligand + excess diazepam) were measured at various concentrations of unlabeled ligand. TB is determined in the absence of any added competitor (non-radioactive ligand) (1, 9). 

*Data analysis*

All of experiments were done in triplicates. The steady state curve was generated by plotting SB versus time in order to determine the proper time of incubation. Receptor concentration curve was generated by plotting [TB/TA] expressed as a percent versus membrane receptor protein concentration. Zone A, the level of membrane protein that yields <10% [TB/TA] was assessed from curve. The saturation curves were generated by plotting the SB versus the radioligand concentration. The binding parameters (Kd and Bmax) of [3H]-Flumazenil were calculated from non-linear regression analysis of the saturation curve data by using the activity base software package (Program Prism, Graph Pad, San Diego, CA). The amount of SB was calculated by subtracting NSB from total binding (TB). TB is the amount of binding of the radioligand in the absence of diazepam. A large excess of diazepam was used in the control experiments to saturate the receptor sites to determine NSB of the radioligand (16-18). 

Competition curve was achieved by plotting the % SB of radioligand versus the increasing concentration of non-radioactive ligand. %SB=[(Bound-NSB)×100]÷(TB-NSB). The percentage inhibition of [3H]-Flumazenil specific binding (IC50) and affinity of non-radioactive ligands for receptors (Ki) were calculated according to Cheng-Prussoff equation (16-18).

## Results and Discussion

**Figure 2:**
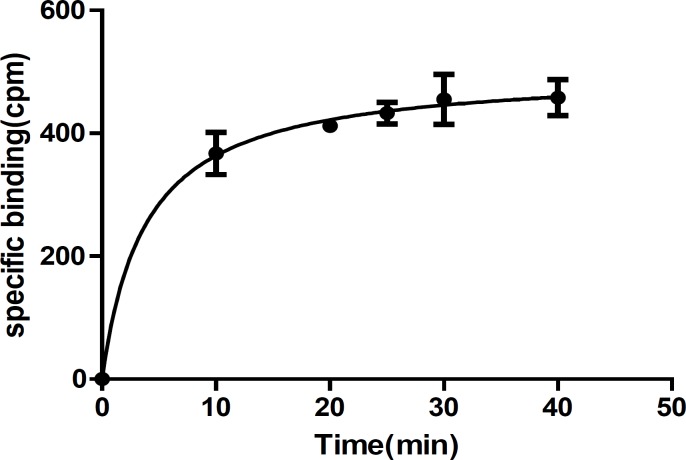
Time course of the specific binding of [3H]-flumazenil to rat cortical membrane benzodiazepine receptors. Each point represents Mean ± SEM of three independent determinations. The line is the best fit to single exponential determined by nonlinear regression analysis

To assess the affinity of newly synthesized ligands against benzodiazepine (BZD) receptors, radioligand receptor binding assays are frequently used. The assays are relatively simple but extremely powerful tool for studying receptors and identify a lead compound for future investigations (9). The assay includes incubation of radioligand with the receptor preparation, separation bound ligand from free ligand, quantify the amount of bound radioligand, and finally analyze the data. The optimum conditions are necessary for experiments in order to get reproducible and reliable results, which are used by researchers to study receptor, analyze the SAR, and finally find a lead compound. Since it is not possible to try out various factors in an attempt to optimize binding, factors such as incubation time and temperature, concentration of radioligand and receptors were evaluated in preliminary studies based on assay conditions by other laboratories. In first attempt, rat cortical membranes were prepared as the source of the BZD receptors. Membrane preparations are most widely used in radioligand receptor binding assays as source of receptors because of reproducibility and reliability of data (1, 14-15). After removal of membrane, the tissue was washed a few times with ice cold buffer to remove any soluble interfering substances such as natural ligand, and guanine nucleotides, which may interfere with the radioligand binding assay (1). Measurement of bound radioligand to membrane preparation containing receptors requires separation of bound from free radioligand, which can be achieved by filtration, centrifugation, or equilibrium dialysis. Based on our previous studies, centrifugation was used to separate bound from unbound ligand (19). Since saturation and competition binding studies are based on equilibrium (theoretical model), thus the time of incubation of ligands and receptors should be enough to ensure that equilibrium or steady state (the time after which the bound no longer increases) has been reached. The time to reach steady state depends on temperature, radioligand and receptor concentration. The incubation can be done on ice, room temperature or at 37 °C. It is most convenient to do the assay at room temperature (1, 14-16). We did the experiment at room temperature, which was measured to be 30 °C. The temperature was kept at 30 °C in all of the experiments. To minimize NSB and prevent ligand depletion, a low concentration of radioligand and membrane preparation is required in assay. Based on literature review, the range of radioligand concentrations should be from (0.1-10) kd if possible (1, 14-16). We obtained the appropriate radioligand concentration in preliminary experiments regarding NSB and reliable measurement of radioactivity (cpm). NSB was determined by excess concentrations of diazepam to occupy all of the available receptors in the presence of radioligand. For most receptor assays a tissue concentration in the range of 100-500 μg of membrane protein is used (1). In preliminary studies, we used 100 μg of membrane protein, which was later proved (in zone A determination) to be the right amount. The appropriate incubation time was determined with incubation of 100 μg of rat cortical membrane contains BZD receptors with the 8.6×10-5 nmole 3H-flumazenil at 30 °C until steady state conditions were reached. The amount of radioligand, which was bound to the receptors at various times after the start of the incubation, was measured. The results were plotted with bound on the Y-axis and time on the X-axis. The appropriate incubation time was determined from the curve where the binding is shown to be constant (1, 14-18). The results of our studies are shown in Table 1 and Figure 2. As can be seen, an incubation time of 35 min is appropriate to reach steady state at 30 °C. 

**Table 1:** The [3H]-flumazenil binding to rat cortical membrane in various time periods

**Incubation time (min)**	**TB (cpm)**	**NSB (cpm)**	**SB (cpm)**
0	0	0	0
10	456.6±60.2	90.3±2.8	367±59.3
20	516±9.8	104±1	412±10.58
25	537±32.5	104.3±2.08	433.6±20.6
30	577.6±79.8	102.3±9.86	455.3±70.4
40	577.6±47.3	99.6±9.29	458±50.06

TB: total binding, NSB: non specific binding, SB: specific binding .

The values shown are the Mean ± SEM of three independent determinations.

**Figure 3:**
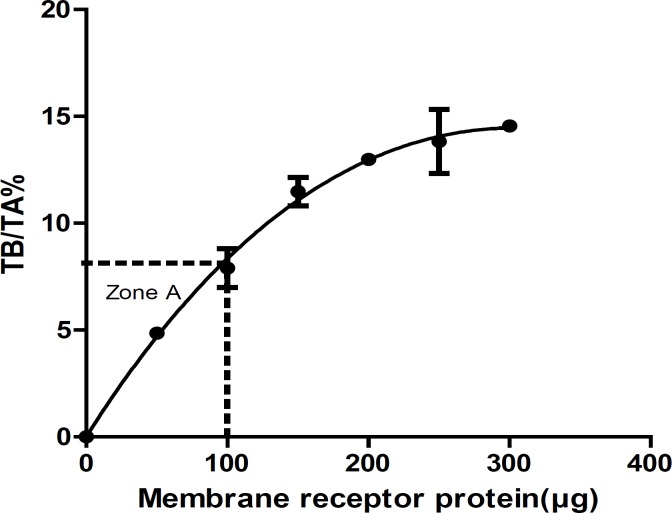
Receptor concentration curve and zone A. Each point represents Mean ± SEM of three independent determinations. The line is the best fit to single exponential determined by nonlinear regression analysis

Receptor concentration**: **In order to obtain the suitable concentration of receptors in the assays, Zone A (the level of receptor that yields <10% TB/TA) must be evaluated from receptor concentration curve by plotting % TB/TA versus receptor concentration. To avoid ligand depletion, the TB of radioligand should be less than 10% of TA radioligand (9, 14-18). Saturation studies must be performed at <10% total ligand binding at all radioligand concentrations. In this study, we measured the amount of TB at various concentrations of receptors in optimum incubation time (35 min). Table 2 summarized the results of receptor concentration. The membrane receptor protein concentration that yields <10% TB/TA (Zone A) was 100 μg (Figure 3). 

**Table 2:** The [3H]-flumazenil binding at various levels of membrane protein in optimum incubation time

**Protein concentration (μg) **	**TB (cpm) **	**TA (cpm) **	**(TB×100)/TA **
0	0	0	0
50	194.3 ± 12.5	3974	4.85 ± 0.27
100	316 ± 61.7	3974	7.9 ± 1.56
150	457.6 ± 46.7	3974	11.48 ± 1.14
200	517.6 ± 14.04	3974	12.98 ± 0.35
250	551.3 ± 105.02	3974	13.83 ± 2.6
300	580 ± 12.28	3974	14.55 ± 0.3

The values shown are the Mean±SEM of three independent determinations

Saturation binding study: For the saturation binding studies of [3H]-flumazenil, seven different concentrations of [3H]-flumazenil (ranging from 0.05 nM to 0.97 nM) were used.

**Figure 4:**
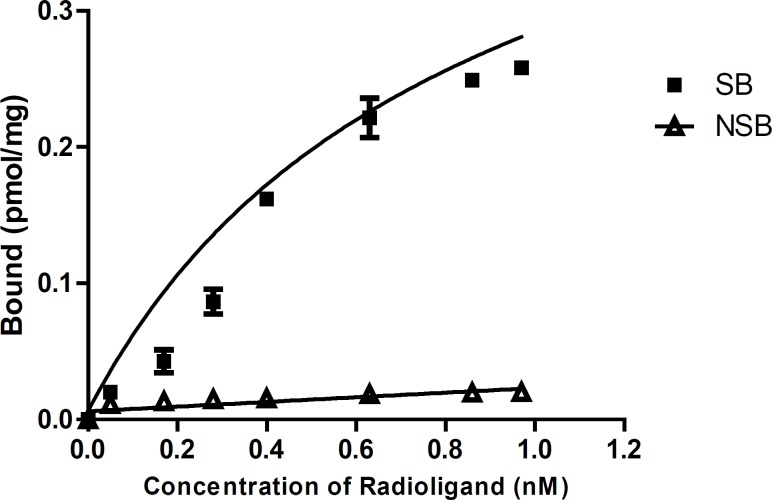
The Saturation curve for the binding of increasing concentrations of [3H]-flumazenil to rat cortical membrane. The amount of radioactivity bound to the tissue, measured in cpm by liquid scintillation counting has been converted to pmol of [3H]-flumazenil per mg of protein present in the incubation mixture.

The amount of radioligand required to saturate the receptors was used to determine the receptor binding affinity of [3H]-Flumazenil (Kd) and the benzodiazepine receptor density (Bmax) based on non-linear regression analysis of the saturation curve data (18). As the concentration of radioligand increases the amount of bound increases until a point is reached where no matter how much more radioligand is added, the amount bound does not increase further. As shown in Table 3 and Figure 4, the binding parameters (Bmax and Kd) of [3H]-Flumazenil were calculated from the saturation binding experiments. Bmax and Kd were calculated as 0.638 ± 0.099 pmol/mg and 1.35 ± 0.316 nM respectively.

**Table 3:** The results of saturation binding at steady state conditions in the presence of seven different concentration of [3H]-flumazeni

**3** **H-flumazenil (nM) **	**TB (cpm) **	**NSB (cpm) **	**SB (cpm) **
0.97	2129.67±11.2	153.5.3±5.3	1976.7±7.7
0.86	2056.6±37.1	149.67±3.53	1907±33.65
0.63	1836±107.3	141.33±3.48	1695±110.6
0.4	1358.33±6.39	118.33±9.83	1240.3±3.93
0.28	776.3±61.9	113.3±8.46	663±69.6
0.17	429±67.1	101.67±5.5	327.36±4.9
0.05	240±16.09	86.67±5.79	153.33±21.4

The values shown are the Mean ± SEM of three independent determinations

Each point represents Mean ± SEM of three independent determinations.

*Competition binding assay*

The affinity of the non-radioactive ligands for the receptor is determined indirectly by measuring their ability to compete and inhibit the binding of radioligand to its receptor. In a competition experiment, various concentrations of a non-radioactive ligand compete with a fixed concentration of radioligand for binding to the receptor. As the concentration of non-radioactive ligand increases, the amount of radioligand bound to the receptor decreases. The concentration of non-radioactive ligand that inhibits the binding of [3H]-flumazenil by 50% is IC50 value (18). 

**Figure 5:**
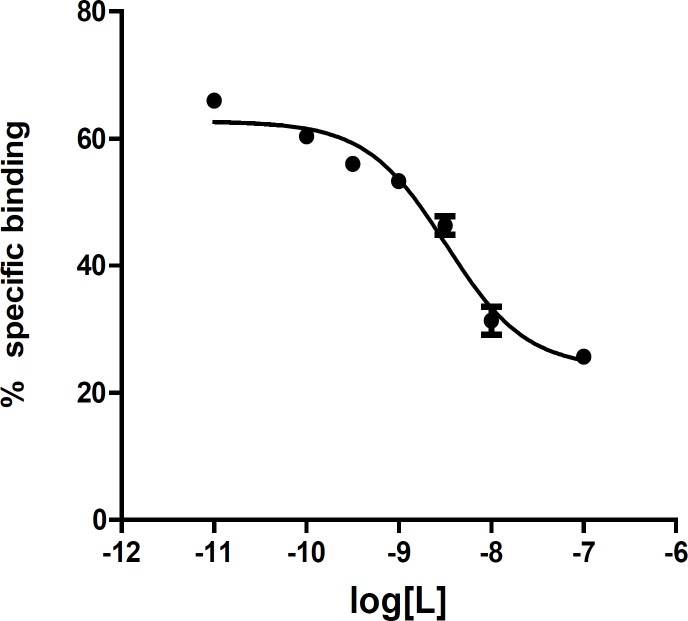
Competition binding curve of 2-phenyl-5-oxo- 7-methyl-1,3,4-oxadiazolo[a,2,3]-pyrimidine as a new benzodiazepine agonist. IC50 curve shows the effect of competitor concentration on the inhibition of radioligand binding. The IC50 value is the concentration of competitor that correlates to the midpoint between the high and low plateaus of the curve.

In this study, the affinity of compound A was measured in competition studies at optimum conditions by displacement of [3H]-Flumazenil from rat cortical membrane (Table 4 and Figure 5). The affinity was 1.9 nM comparable with diazepam (1.53 nM), a known benzodiazepine agonist. This finding makes the compound an interesting lead for further optimization. Starting from this compound, new ligands were designed and synthesized. The design was based on a pharmacophore model of the benzodiazepine binding site of GABAA. The affinity and IC50 of new compounds were measured in competition studies. The *in-vivo *biological evaluation of compounds with good affinity is on the way. The results (design, synthesis, and biological evaluation) would be published soon. 

In conclusion, radioligand receptor binding assays at optimum conditions provide *in-vitro *screening of compounds quickly and precisely. Compounds with high affinity would go through biological evaluation. Based on receptor structure, essential pharmacophore groups, and affinity of ligands, a lead compound would be identified.

**Table 4:** The results of competition experiment of 2-phenyl-5-oxo-7-methyl-1,3, 4-oxadiazolo[a,2,3]-pyrimidine in the presence of increasing concentration of [3H]-flumazenil

log[L*]	%Specific binding
-11.0	66 ± 1
-10.0	60 ± 1.5
-9.5	56 ± 1.7
-9.0	53 ± 1.5
-8.5	46 ± 2.5
-8.0	31 ± 3.7
-7.0	25 ± 0.5

The values shown are the Mean±SEM of three independent determinations

Each point represents Mean ± SEM of three independent determinations.
